# Efficient Vibration Measurement and Modal Shape Visualization Based on Dynamic Deviations of Structural Edge Profiles

**DOI:** 10.3390/s24134413

**Published:** 2024-07-08

**Authors:** Andong Zhu, Xinlong Gong, Jie Zhou, Xiaolong Zhang, Dashan Zhang

**Affiliations:** 1 College of Engineering, Anhui Agricultural University, Hefei 230036, China; zhuad@stu.ahau.edu.cn (A.Z.); ahaugxl@stu.ahau.edu.cn (X.G.); zhoujie@stu.ahau.edu.cn (J.Z.); xlzhang@ahau.edu.cn (X.Z.); 2 Intelligent Agricultural Machinery Laboratory of Anhui Province, Anhui Agricultural University, Hefei 230036, China

**Keywords:** vision-based measurement, experimental modal analysis, dynamic deviation extraction, modal shape visualization, high-speed camera system

## Abstract

As a non-contact method, vision-based measurement for vibration extraction and modal parameter identification has attracted much attention. In most cases, artificial textures are crucial elements for visual tracking, and this feature limits the application of vision-based vibration measurement on textureless targets. As a computation technique for visualizing subtle variations in videos, the video magnification technique can analyze modal responses and visualize modal shapes, but the efficiency is low, and the processing results contain clipping artifacts. This paper proposes a novel method for the application of a modal test. In contrast to the deviation magnification that exaggerates subtle geometric deviations from only a single image, the proposed method extracts vibration signals with sub-pixel accuracy on edge positions by changing the perspective of deviations from space to timeline. Then, modal shapes are visualized by decoupling all spatial vibrations following the vibration theory of continuous linear systems. Without relying on artificial textures and motion magnification, the proposed method achieves high operating efficiency and avoids clipping artifacts. Finally, the effectiveness and practical value of the proposed method are validated by two laboratory experiments on a cantilever beam and an arch dam model.

## 1. Introduction

Structural modal characteristics, including modal frequencies, damping ratios, and modal shapes, are crucial parameters in structural health monitoring (SHM) and non-destructive testing (NDT) [1,2,3,4,5]. For applications of failure estimation and topological optimization, the measurement requirements of these properties are widely presented in various scales, from micro-nano to construction structures. Researchers have made great efforts to acquire critical data from structural dynamic responses for these properties. The most common types of sensors in modal experiments include the accelerometer, Laser Doppler Vibrometer (LDV), and strain gauge [6,7,8]. Although observations relying on these sensors are accurate and reliable, the spatial resolution in measurement is limited by the quantity of sensors.

Vision-based measurement techniques provide an alternative for modal testing with a high spatial resolution [9,10,11,12,13]. As a non-contact method, vision-based devices are easy to install and provide higher resolution in space. Combined with image processing algorithms, such as the image registration [14,15] and digital image correlation (DIC) [16,17,18], vibration or deformation can be estimated through relative pixel shift. In most cases, artificial textures like speckles and markers are crucial elements for tracking and locating, which have a significant impact on the quality of measuring. This feature limits the application of vision-based vibration measurement for textureless engineering targets, especially in the outdoor environment.

In recent years, the video magnification technique has been developed and introduced into experimental modal tests [19,20,21,22,23,24]. By connecting spatial vibration to temporal variation, video magnification algorithms extract vibration signals from intensity/phase variations in the timeline. Then, structural modal parameters such as the natural frequencies and damping ratios can be identified from the vibration data. Also, modal shapes can be visualized by exaggerating spatial vibrations corresponding to a specific modal order. Studies have verified the applicability of video magnification algorithms in vibration tests on various engineering structures, such as arch dams, buildings, bridges, etc. [25,26,27,28,29]. Most video magnification frameworks provide full-field measurement capability without requiring a speckle or marker. However, the manipulation of spatial features is usually complicated and tedious. For instance, in the phase-based Eulerian video magnification (PEVM) [19], vibration signals are extracted from temporal phase variations decomposed by spatial filters. Meanwhile, the increasing of spatial filters will not only reduce the clipping artifacts and image blurs in the final motion magnification but also significantly increase the burden on the processor and memory.

To enhance the efficiency of vision-based vibration tests, this paper proposes a vibration extraction and modal shape analysis method based on dynamic deviations on structural edge profiles. In contrast to the deviation magnification [30] that exaggerates subtle geometric deviations from only a single image, the proposed method extracts vibration signals with sub-pixel accuracy on edge positions by turning the perspective of deviations from space to timeline. Then, according to the vibration theory of continuous linear systems, structural modal shapes are visualized using spatial weights on edge positions. Without involving spatial filters and motion magnification, the proposed method has a compact structure and achieves high operating efficiency.

The main contributions of this paper are summarized as follows: (1) An efficient vibration extraction and modal shape analysis method is proposed based on dynamic deviations. (2) The relationship between temporal deviation and spatial weights is detailed, and the main technical issues are discussed. (3) The performance of the proposed method is validated by two modal experiments on a beam structure and an arch structure.

The rest of this paper is organized as follows: Section 2.1 and Section 2.2 introduce the vibration extraction theory based on temporal deviations and discuss the pixel sampling issue of unilateral and bilateral edges, respectively. A simulation test is also presented in Section 2.2 to better understand the whole process and validate signal accuracy. Section 2.3 describes the relationship between the temporal deviations and linear vibration system in detail and presents the temporal and spatial processing in visualizing modal shapes. In Section 3.1, two modal experiments on a cantilever beam and an arch dam model are given to further evaluate the performance of the proposed method. Finally, Section 4 and Section 5 provide the discussion and conclusion of this paper.

## 2. Materials and Methods

In the original deviation magnification algorithm, the variations along a canonical stripe in a single image are the prerequisites for generating deformation fields and achieving magnification [30]. In this study, by changing the perspective of deviations from space to the timeline, temporal deviations along the edges can be estimated in the same way, and vibration signals on entire edge coordinates can be effectively extracted. For simplicity, the methodology section only discusses the situation of the linear edge. This method can also be applied to more complicated situations, such as the ellipse (circle) and other high-order curves after temporal sampling along the normal directions.

### 2.1. Dynamic Deviations and Vibration

Consider a synthetic image $I\left(t,x,y\right)$, which has a linear edge along the *x*-axis and vibrates only in the *y*-direction, the intensity values along the *n*-th column in the timeline is $I_{x_{n}}\left(t,y\right)$. Define temporal variations in $I\left(t,x,y\right)$ at position $x_{n}$ and time *t* as

(1) $$S_{x_{n},t}\left(y\right):=I_{x_{n}}\left(t,y\right).$$

The vibration signal at position $x_{n}$ is closely related to deviations in $S_{x_{n},t}\left(y\right)$.

Assuming there is no vibration (i.e.,∀t$f_{x_{n}}\left(t\right)=0$) at position $x_{n}$, the temporal variations along $I_{x_{n}}\left(t,y\right)$ will be constant, i.e., $S_{x_{n},t}\left(y\right)=S_{x_{n}}\left(y\right)$. Given the vibration signal $f_{x_{n}}\left(t\right)$ at position $x_{n}$, the relationship between $f_{x_{n}}\left(t\right)$ and $S_{x_{n},t}\left(y\right)$ can be represented as:

(2) $$S_{x_{n},t}\left(y\right)=S_{x_{n}}\left(y+f_{x_{n}}\left(t\right)\right).$$

Then, the vibration extraction at $x_{n}$ turns into estimating $f_{x_{n}}\left(t\right)$ given the observations of $S_{x_{n},t}\left(y\right)$.

In practice, $S_{x_{n}}\left(y\right)$ can be computed by aggregating information from all available spatial-temporal slices at $x_{n}$. Since $f_{x_{n}}\left(t\right)$ usually has a small value, the mean of the spatial-temporal slices can be exploited to calculate $S_{x_{n}}\left(y\right)$. Assuming that the noise is independent in the timeline, the sampled $I_{x_{n}}\left(t,y\right)$ is represented as:

(3) $$I_{x_{n}}\left(t,y\right)=S_{x_{n}}\left(y+f_{x_{n}}\left(t\right)\right)+g_{x_{n}}\left(t,y\right),$$

where $g_{x_{n}}\left(t,y\right)$ denotes the temporal noise. Then, using a first-order Taylor expansion of $S_{x_{n}}\left(y+f_{x_{n}}\left(t\right)\right)$ leads to:

(4) $$I_{x_{n}}\left(t,y\right)\approx S_{x_{n}}\left(y\right)+f_{x_{n}}\left(t\right)S_{x_{n}}^{\prime }\left(y\right)+g_{x_{n}}\left(t,y\right).$$

Thus, the mean over *t* is:

(5) $$\frac{1}{D_{t}}\sum_{t}I_{x_{n}}\left(t,y\right)\approx S_{x_{n}}\left(y\right)+\mu_{f}S_{x_{n}}^{\prime }\left(y\right)+\frac{1}{D_{t}}\sum_{t}g_{x_{n}}\left(t,y\right),$$

where $\mu_{f}$ denotes the mean of $f_{x_{n}}\left(t\right)$ over *t*, and $D_{t}$ represents the number of frames in the *t* direction. Since $\mu_{f}$ also has a small value, using the Taylor expansion again, we have:

(6) $$S_{x_{n}}\left(y\right)+\mu_{f}S_{x_{n}}^{\prime }\left(y\right)\approx S_{x_{n}}\left(y+\mu_{f}\right).$$

Thus, the average temporal variation can approximate the common variation up to a constant shift of $\mu_{f}$. After the value of $S_{x_{n}}\left(y\right)$ is obtained, $f_{x_{n}}\left(t\right)$ is calculated in terms of the least square error between the $S_{x_{n}}\left(y\right)$ and each of the observed ones:

(7) $$arg\min\sum_{y}{\left(I_{x_{n}}\left(t,y\right)-S_{x_{n}}\left(y\right)-f_{x_{n}}\left(t\right)S_{x_{n}}^{\prime }\left(y\right)\right)}^{2},$$

which leads to the vibration signal at position $x_{n}$:

(8) $$f_{x_{n}}\left(t\right)\approx \frac{\sum_{y}\left(I_{x_{n}}\left(t,y\right)-S_{x_{n}}\left(y\right)\right)S_{x_{n}}^{\prime }\left(y\right)}{\sum_{y}S_{x_{n}}^{\prime }{\left(y\right)}^{2}}$$

The above equation indicates that the pixels for which $S_{x_{n}}^{\prime }\left(y\right)=0$ do not affect the solution. By sampling spatial-temporal slices at all edge coordinates, vibration signals can be extracted by continually computing the dynamic deviations.

### 2.2. Unilateral and Bilateral Samplings

From the derivations above, it can be seen that the vibration extraction at $x_{n}$ depends on the temporal sampling result $I_{x_{n}}\left(t,y\right)$. For those structures with thin cross-sections, such as the trusswork and arch structure, temporal sampling on each side of the edge almost doubles the amount of *n* and requires additional control of the pixel amount in the *y* direction. Thus, bilateral sampling will be more convenient.

Assuming that the bilateral sides of the structure are mirror-symmetric with respect to a centerline, by Equation (8), the vibration extraction result depending on bilateral sampling can be represented as:

(9) $${\hat{f}}_{x_{n}}\left(t\right)\approx \frac{\sum_{y}\left(\left(\begin{array}{c} I_{x_{n}}\left(t,y\right)\\ MI_{x_{n}}\left(t,y\right) \end{array}\right)-\left(\begin{array}{c} S_{x_{n}}\left(y\right)\\ MS_{x_{n}}\left(y\right) \end{array}\right)\right)\left(\begin{array}{c} S_{x_{n}}^{\prime }\left(y\right)\\ MS_{x_{n}}^{\prime }\left(y\right) \end{array}\right)}{\sum_{y}{\left(\left(\begin{array}{c} S_{x_{n}}^{\prime }\left(y\right)\\ MS_{x_{n}}^{\prime }\left(y\right) \end{array}\right)\right)}^{2}}$$

where ${\hat{f}}_{x_{n}}\left(t\right)$ denotes the vibration signal, and M is the mirror-symmetric operator (diagonal matrix). Obviously, from Equations (8) and (9), $f_{x_{n}}\left(t\right)$ and ${\hat{f}}_{x_{n}}\left(t\right)$ are equal when the differences between each side of the edge are ignored.

**Figure 1:**
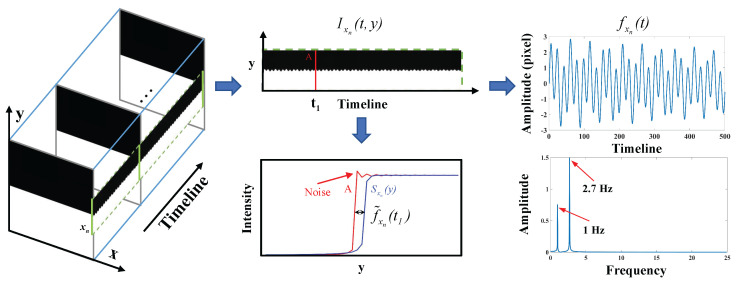
A simulation for understanding how to extract the dynamic deviations through the edge profile.

**Figure 2:**
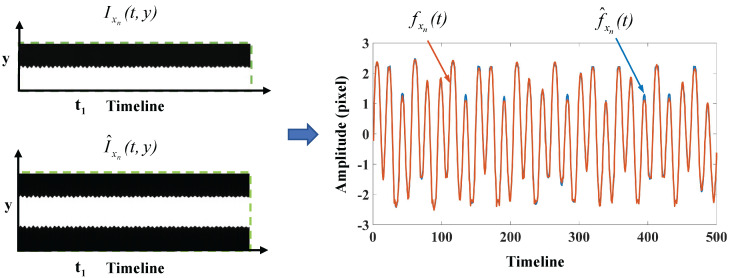
Comparison of the vibration signals $f_{x_{n}}\left(t\right)$ and ${\hat{f}}_{x_{n}}\left(t\right)$.

Here, a simulation test is conducted to better understand the vibration extraction process and validate its accuracy. As illustrated in Figure 1, an image with a clear dividing line is programmed to vibrate by the phase shifting (only in the *y* direction) in a synthetic damped sinusoidal manner as:

(10) $$\delta \left(t\right)=\xi_{1}sin\left(2\pi f_{1}t\right)e^{\left(-\alpha_{1}t\right)}+\xi_{2}sin\left(2\pi f_{2}t\right)e^{\left(-\alpha_{2}t\right)}.$$

where $\xi_{1}$=1, $\xi_{2}$=2, $f_{1}$=1Hz, $f_{2}$=2.7Hz, and both $\alpha_{1}$ and $\alpha_{2}$ are 0.03. The sampling position $x_{n}$ is set to the right side of the image. After temporal sampling at position $x_{n}$, the deviations of $I_{x_{n}}\left(t,y\right)$ reflect vibration $f_{x_{n}}\left(t\right)$. Through analysis, the Root Mean Square Error (RMSE) between $\delta \left(t\right)$ and $f_{x_{n}}\left(t\right)$ is 0.1927. As demonstrated in Figure 2, the comparison of the vibration signals between $f_{x_{n}}\left(t\right)$ and ${\hat{f}}_{x_{n}}\left(t\right)$ indicates that there is little difference in the vibration signals extracted using unilateral sampling and bilateral sampling. The RMSE between $f_{x_{n}}$ and ${\hat{f}}_{x_{n}}\left(t\right)$ is 0.2119. It is worth noting that the phase-shifting of images will introduce noise to the edge (shown in Figure 1), making the vibration signals affected by temporal and spatial noise.

### 2.3. Estimation of Spatial Weight

After calculating vibration signals on the edge, the spatial weight is then estimated. According to the theory of the modal superposition method, the structural, spatial vibration $f_{x_{n}}\left(t\right)$ is a linear combination of modal responses for a Rayleigh damping system, and it can be approximated by the edge vibration signal:

(11) $$f_{x_{n}}\left(t\right)=\sum_{i=1}^{k}\phi_{i}\left(x_{n}\right)q_{i}\left(t\right)e^{\left(-\alpha_{i}t\right)}.$$

where *k* denotes the maximum mode order being excited, $\phi_{i}\left(x_{n}\right)$ represents the weight corresponding to the *i*-th mode at position $x_{n}$, $q_{i}\left(t\right)$ denotes the *i*-th temporal modal response, and the $\alpha_{i}$ is the attenuation coefficient. Considering vibration signals on all sampling positions, the relationship between spatial weights, modal responses, and vibration signals is expressed as follows:

(12) $$\left[\begin{array}{cccc} \phi_{1}\left(x_{1}\right) & \phi_{2}\left(x_{1}\right) & \cdots & \phi_{k}\left(x_{1}\right)\\ \phi_{1}\left(x_{2}\right) & \phi_{2}\left(x_{2}\right) & \cdots & \phi_{k}\left(x_{2}\right)\\ \vdots & \vdots & \vdots & \vdots \\ \phi_{1}\left(x_{n}\right) & \phi_{2}\left(x_{n}\right) & \cdots & \phi_{k}\left(x_{n}\right)\\ \vdots & \vdots & \vdots & \vdots \\ \phi_{1}\left(x_{N}\right) & \phi_{2}\left(x_{N}\right) & \cdots & \phi_{k}\left(x_{N}\right) \end{array}\right]\left[\begin{array}{c} e^{\left(-\alpha_{1}t\right)q_{1}\left(t\right)}\\ e^{\left(-\alpha_{2}t\right)q_{2}\left(t\right)}\\ \vdots \\ e^{\left(-\alpha_{i}t\right)q_{i}\left(t\right)}\\ \vdots \\ e^{\left(-\alpha_{k}t\right)q_{k}\left(t\right)} \end{array}\right]\approx \left[\begin{array}{c} f_{x_{1}}\left(t\right)\\ f_{x_{2}}\left(t\right)\\ \vdots \\ f_{x_{n}}\left(t\right)\\ \vdots \\ f_{x_{N}}\left(t\right) \end{array}\right].$$

**Figure 3:**
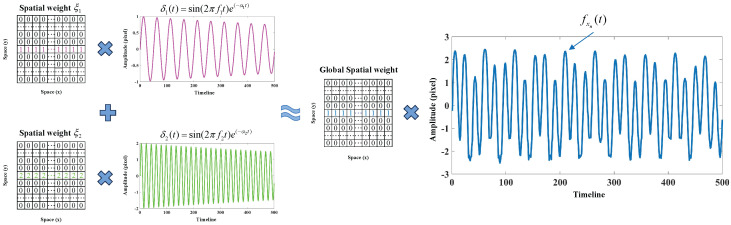
The representation of spatial motion based on the theory of linear vibration systems in the simulation test.

It can be seen from the equation that each column of the first bracket on the left side represents the spatial weights corresponding to a specific vibration mode, and each column of the second bracket represents the modal response of each order. Since $f_{x_{n}}\left(t\right)$ can be observed, the estimation of spatial weights on all edge positions mainly depends on the modal responses [28]. The modal superposition theory is first validated through the above simulation test. As illustrated in Figure 3, the programmed signal $\delta \left(t\right)$ is a combination of two independent damped vibrations. In this simulation, all spatial weights on the edges are constant values ($\xi_{1}$ and $\xi_{2}$).

#### 2.3.1. Temporal Processing

It can be seen from the above introduction that the calculation of spatial weights requires the estimation of modal responses. In linear vibration theory, structural responses in the modal test are usually considered to be combinations of damped signals of each mode. To realize an efficient process, the variational mode decomposition (VMD) algorithm is used in this study to estimate modal responses [31]. As an efficient self-adaption signal processing method, the VMD decomposes the raw signal into a sequence of intrinsic mode functions (IMFs) by solving a variational problem. This algorithm has been verified in separations of mixed vibration signals [32,33].

For an observed $f_{x_{n}}\left(t\right)$, the structural modal response of each order $e^{\left(-\alpha_{i}t\right)}q_{i}\left(t\right)$ can be approximated by decomposed modal components $h_{v}\left(t\right)$:

(13) $$\left\{\begin{array}{c} \underset{\left\{h_{v}\left(t\right)\right\},\left\{\omega_{v}\right\}}{min}=\left\{\sum_{v=1}^{V}{∥\partial \left(t\right)\left[\left(\eta \left(t\right)+j\frac{1}{\pi t}\right)*h_{v}\left(t\right)\right]e^{-j\omega_{v}t}∥}_{2}^{2}\right\}\\ s.t.\sum_{v=1}^{V}h_{v}\left(t\right)=f_{x_{n}}\left(t\right) \end{array}\right.$$

where $\omega_{v}$ denotes the center frequency, *V* denotes the maximum mode number being disassembled, ∂(t) represents the partial derivative with time *t*, η is the Dirac distribution, * denotes the convolution operator, $\left\{h_{v}\left(t\right)\right\}=\left\{h_{1}\left(t\right),h_{2}\left(t\right),\cdots ,h_{V}\left(t\right)\right\}$ represents the mode ensemble, and $\left\{\omega_{v}\right\}=\left\{\omega_{1},\omega_{2},\cdots ,\omega_{V}\right\}$ denotes the corresponding center frequency ensemble. The constraint is that the sum of the modes is equal to the original signal $f_{x_{n}}\left(t\right)$.

To solve the above optimization problem, a quadratic penalty term and a Lagrangian multiplier are introduced to transform it into the following unconstrained problem:

(14) $$\begin{array}{cc} \; & L\left(\left\{h_{v}\left(t\right)\right\},\left\{\omega_{v}\right\},\lambda \right)=\beta \sum_{v=1}^{V}{∥-j\omega_{v}\left[\left(\eta \left(t\right)+j\frac{1}{\pi t}\right)*h_{v}\left(t\right)\right]e^{-j\omega_{v}t}∥}_{2}^{2}\\ \; & +{∥f_{x_{n}}\left(t\right)-\sum_{v=1}^{V}h_{v}\left(t\right)∥}_{2}^{2}+\langle \lambda \left(t\right),f_{x_{n}}\left(t\right)-\sum_{v=1}^{V}h_{v}\left(t\right)\rangle \end{array}$$

where β is the penalty parameter, and λ is the Lagrangian multiplier. The mode ensemble $\left\{h_{v}\left(t\right)\right\}$ can approximate representation $e^{\left(-\alpha_{i}t\right)}q_{i}\left(t\right)$.

In practice, VMD can be easily implemented by calling the MATLAB inbuilt functions. If the input vibration signal is not selected from the stationary points of a structure, the modal responses can be estimated without prior knowledge of the intrinsic frequencies. It is noteworthy that the amplitude of the estimated modal responses affects only the global scale of spatial weights [28]. Nevertheless, it is still recommended to normalize them before applying them to Equation (12).

#### 2.3.2. Spatial Processing

After the system responses are obtained, the spatial weights of each modal shape can be calculated by solving Equation (12). Considering that the $\phi_{i}\left(x\right)$ in high-frequency regions is not meaningful, the operation in space focuses on removing high-frequency components. Since spatial weights on continuous edges can be regarded as 1D signals, moving-average filtering (MAF) is used in this study to improve the quality of each set of spatial weights [34]. As a simple low-pass finite impulse response filter, the MAF smoothes the sampled data and replaces the original data with the average of the neighboring points within a defined scaling range. The smoothed spatial weights are expressed as:

(15) $${\tilde{\phi }}_{i}\left(x_{n}\right)=\frac{1}{m}\sum_{u=0}^{m-1}\phi_{i}\left(x_{n+u}\right)$$

where ${\tilde{\phi }}_{i}\left(x\right)$ denotes the smoothed weight of each modal response, and *m* is the number of points used in the moving average. By plotting the smoothed weight ${\tilde{\phi }}_{i}\left(x\right)$, the structural modal shapes can be observed.

## 3. Results

To validate the effectiveness of the proposed method, two laboratory modal tests on a beam structure and arch structure are provided in the experiment section. The technical information about the Chronos 2.1-HD high-speed camera system is listed in Table 1. All elapsed times of these two experiments are computed on MATLAB R2020b on a laptop equipped with a single AMD Ryzen 7 5800H processor (3.20 GHz).

### 3.1. Experiment of the Beam

**Figure 4:**
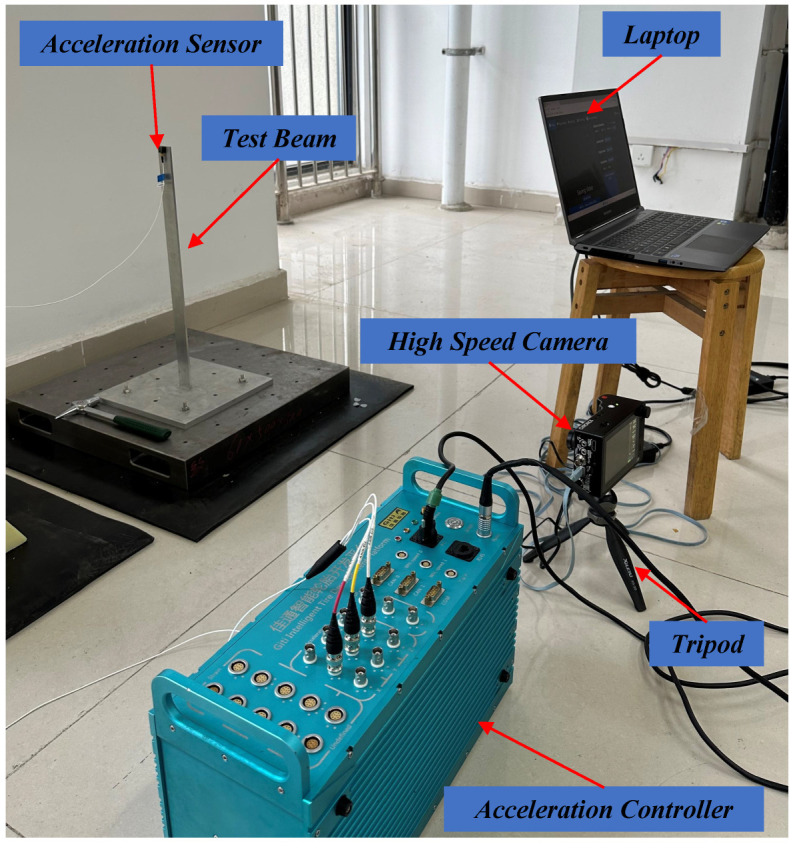
The experimental setup for the beam experiment.

As illustrated in Figure 4, a beam was installed on the metal base and then driven by an impact excitation at the bottom by a force hammer. The aluminum alloy beam has a dimension of 500 mm × 30 mm × 5 mm. The vibration of the test beam was recorded by the Chronos 2.1-HD high-speed camera at 1069 frames per second (fps) with a resolution of 1280 × 1024 pixels. The vibration video of the beam was captured with a duration of up to 8 s. The vibration signals were also collected by an accelerometer placed at the top of the beam. The accelerometer of type DYTRAN/3333MT weighs about 4 g, so its additional mass effect on the beam can be almost negligible.

**Figure 5:**
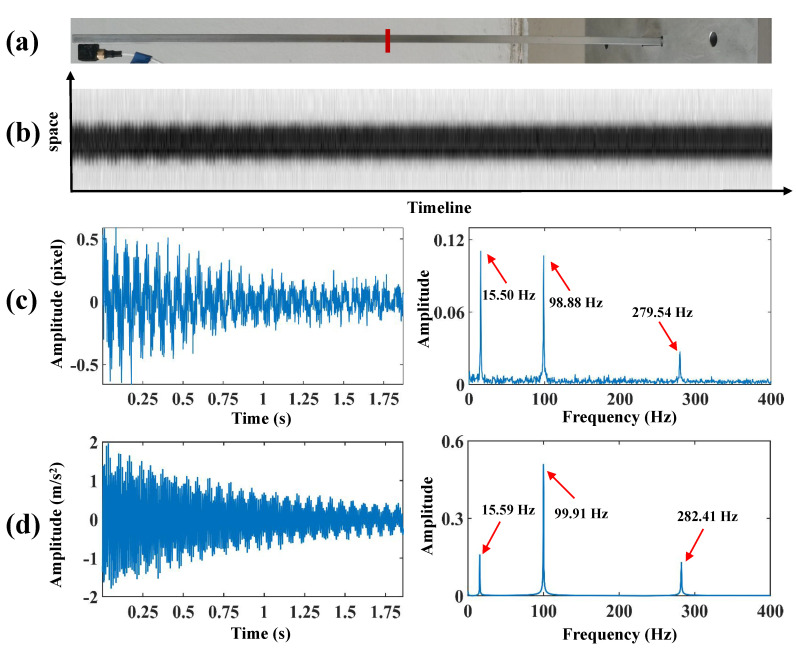
(**a**) The location of the spatial-temporal slice in the beam test, (**b**) the spatial-temporal slices of bilateral edges, (**c**) the vibration signal from bilateral edges and frequency spectra, (**d**) the vibration signal from the accelerometer and frequency spectra.

**Figure 6:**
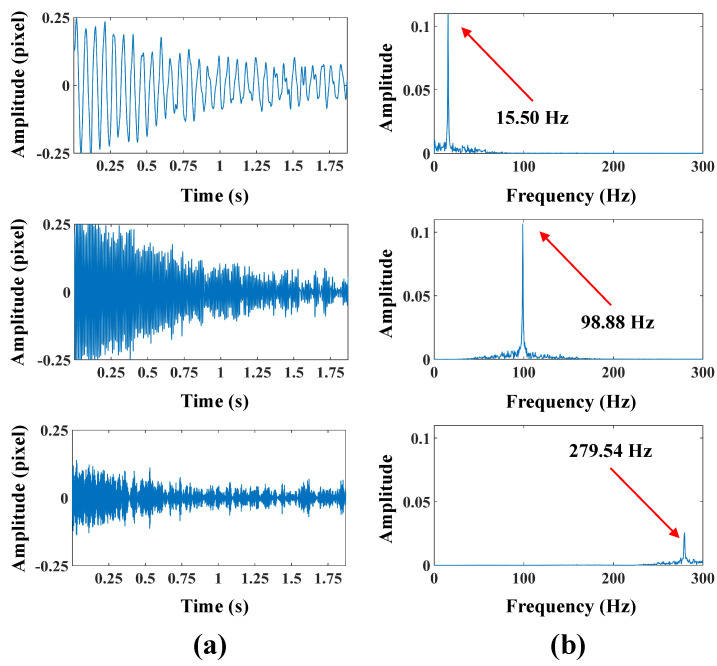
(**a**,**b**), first three orders of the modal responses and frequency spectra in the beam test.

The location of the spatial-temporal slice in the beam test is demonstrated in Figure 5a. Figure 5b shows the spatial-temporal slices of bilateral edges, and the vibrational signals of the structure are characterized by irregular edges. Figure 5c demonstrates the vibration signal from bilateral edges and frequency spectra, and it can be observed that the extracted vibration signals reach sub-pixel level accuracy. Figure 5d shows the vibration signal from the accelerometer and frequency spectra. Then, the modal responses are recovered by the VMD algorithm. The separated first three orders of modal responses and frequency spectra are presented in Figure 6. Also, three obvious peaks, including 15.50 Hz, 98.88 Hz, and 279.54 Hz, are detected from their frequency spectra. The parameters of the beam and analysis of the modal frequencies in the beam test are listed in Table 2. The errors between the finite element simulation and experimental modal frequencies suggest that the proposed method can accurately extract the modal responses in the beam test. To estimate the spatial modal shapes of the beam, the vibration signals of all the columns that make up the edge of the beam must be extracted. The edge of the beam in the image consists of 550 columns of pixels; 2000 frames are selected from the video and processed to extract the vibration signals from these 550 columns, and the whole process consumes only 35.6 s.

**Figure 7:**
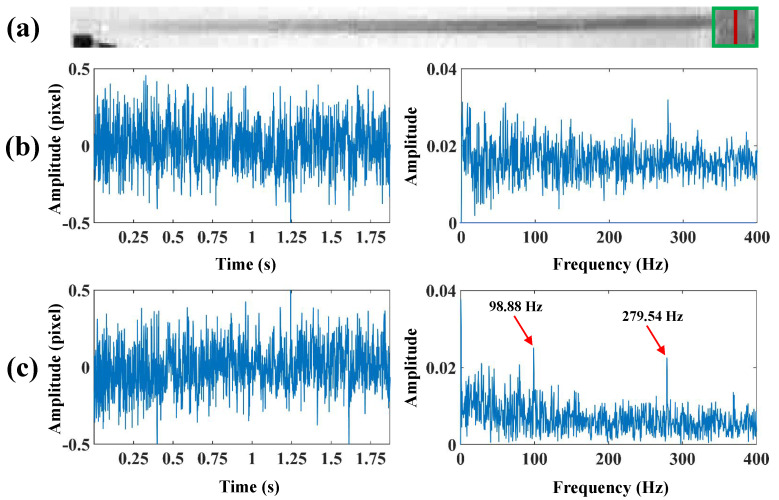
(**a**) The grayscale image of the beam, (**b**) the vibration signal extracted before sharpening and frequency spectra, (**c**) the vibration signal extracted after sharpening and frequency spectra.

**Figure 8:**
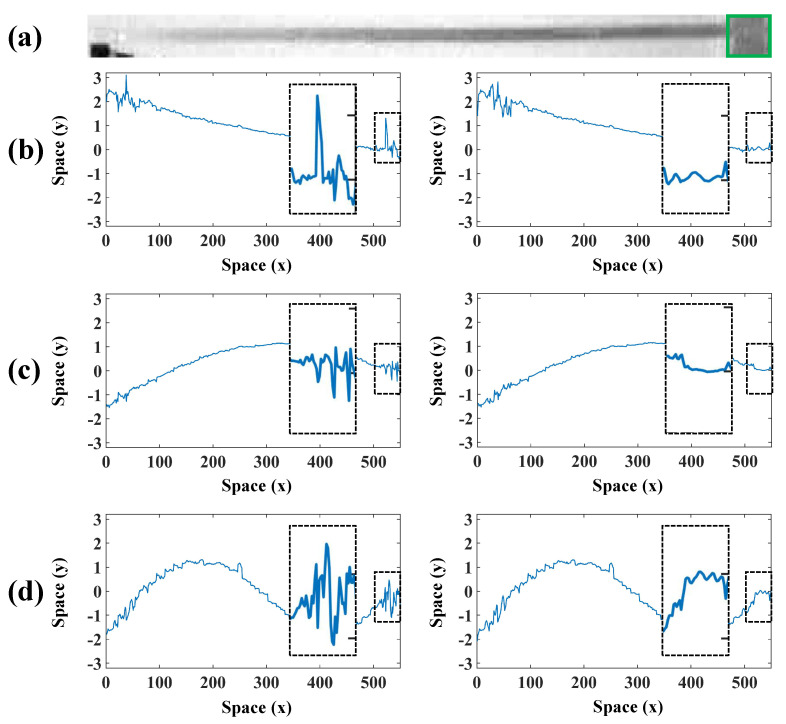
(**a**) The grayscale image of the beam, (**b**–**d**) comparison of the first three orders of the spatial weights before and after sharpening in the beam test.

**Figure 9:**
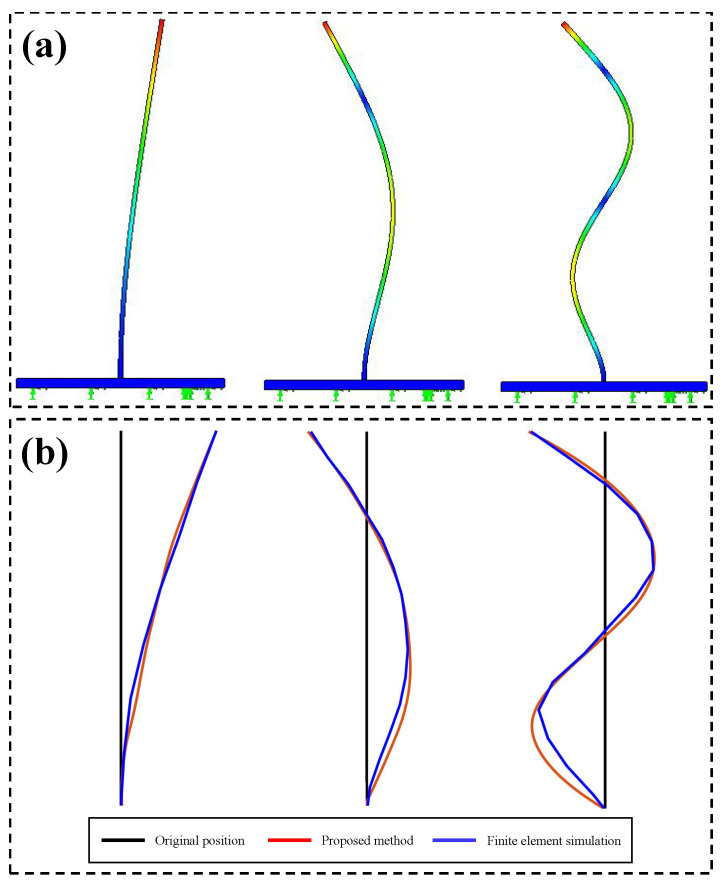
(**a**) The modal shapes obtained by finite element simulation in the beam test, (**b**) comparison of the normalized modal shapes obtained by finite element simulation and the proposed method in the beam test.

As illustrated in Figure 7a, the bottom of the beam marked with a green box is affected by light and background interference, which in turn affects the accuracy of the extracted vibration signals. Considering that the edges of the beam are horizontal in the image, a horizontal sharpening convolution kernel is used at the bottom of the beam image to improve the quality of the edges. Figure 7b,c present the comparison of the vibration signals extracted from the bottom of the beam and spectra before and after sharpening, and it can be seen that more valuable vibration signals can be extracted from the sharpened image. Figure 8 compares the spatial weights before and after sharpening in the beam test. It can be observed that the spatial weights at the bottom of the beam extracted from the sharpened image are smoother and more accurate. Then, the spatial weights are smoothed using the MAF, and the modal shapes can be plotted based on the smoothed spatial weights. The modal shapes obtained by finite element simulation in the beam test are illustrated in Figure 9a. Figure 9b shows the comparison of the normalized modal shapes obtained by finite element simulation and the proposed method in the beam test. Since there are differences in material property, constraint conditions, and experimental control between the actual experiment and finite element simulation under ideal conditions, deviations can be observed in the comparison results. As shown in Figure 9, for a rigid structure with bilateral parallel sides, the spatial vibration shapes at the edges can be exploited to characterize the structural modal shapes. The accuracy of modal shapes obtained from the proposed method is further quantitatively validated by means of the cross-modal assurance criterion (cross-MAC) values [35], provided in Table 3. The cross-MAC values are close to one, therefore demonstrating the precision of the proposed method when compared to conventional finite element simulation.

### 3.2. Experiment of the Arch Dam Model

Arch dams transmit a large portion of the water pressure and other loads through thrust to the abutment, exploiting the compressive strength of its material. Therefore, their design and construction require sophisticated engineering knowledge. Since water considerably affects the dynamic response of arch dams, regular inspections are necessary to identify potential problems and reduce these risks. One of the important inspections is to apply vibration tests to arch dams to detect damage by investigating changes in the dynamic characteristics, mainly changes in the modal frequencies and modal shapes [36,37].

**Figure 10:**
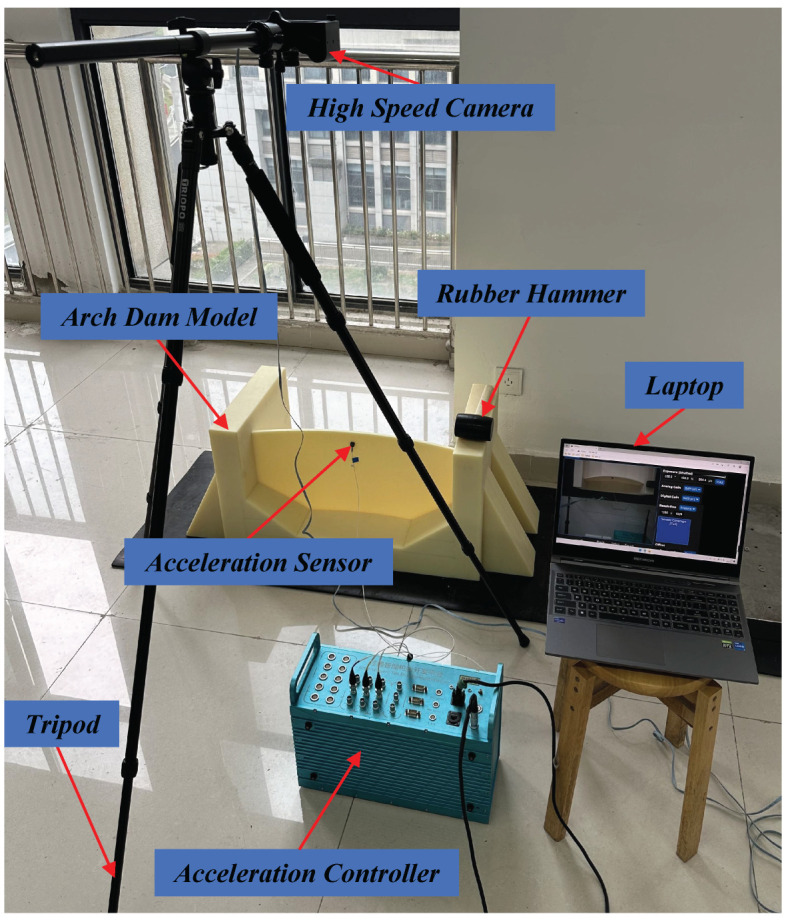
The experimental setup for the arch dam model test.

To extract the modal responses and modal shapes of arch dams, the proposed method was performed on an arch dam model made of plastic. As demonstrated in Figure 10, the arch dam model consists of the dam base and dam body, and it was placed on an anti-slip mat. In the experiment, an impact excitation was given to excite the arch dam model on the dam body using a force hammer, and the vibration of the test structure was recorded by the Chronos 2.1-HD high-speed camera at 1069 fps with a resolution of 1280 × 1024 pixels. The vibration video of the arch dam model was captured with a duration of up to 8 s. Meanwhile, the vibration signals were also collected by an accelerometer placed at the top of the dam body.

Since the arch dam model is a circular structure, the pixel coordinates of the circle center $\left(x_{0},y_{0}\right)$ and radius $r_{0}$ can be located using Hough circles. Then, the coordinates of the pixels that make up the arch dam model are calculated following Equation (16):

(16) $$\left\{\begin{array}{c} x_{p}=x_{0}+r_{0}cos\left(p\theta \right)\\ y_{p}=y_{0}+r_{0}sin\left(p\theta \right) \end{array}\right.,$$

where $\left(x_{p},y_{p}\right)$ are the pixel coordinates of arch dam model, *p* denotes the serial number of pixel, and θ denotes the angle between adjacent pixels.

**Figure 11:**
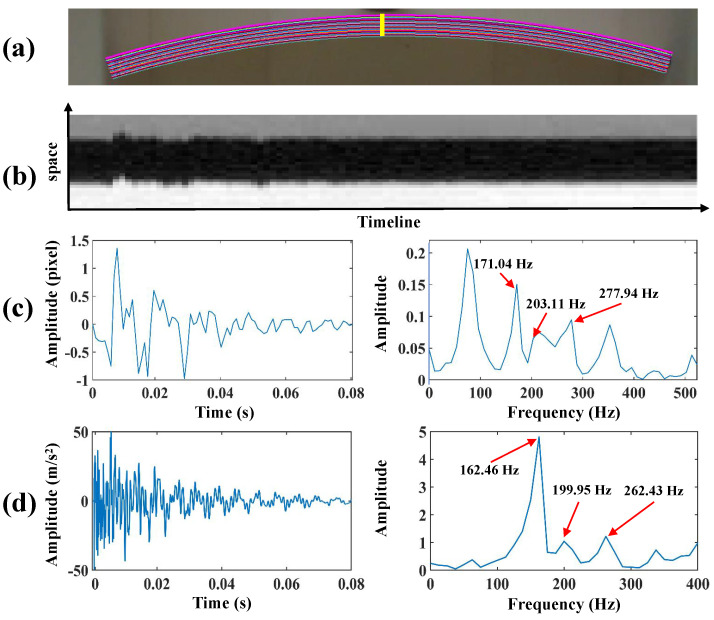
(**a**) The extraction results of the dam body pixels and the location of the spatial-temporal slice in the arch dam model test, (**b**) the spatial-temporal slices from bilateral edges, (**c**) the vibration signal from bilateral edges and frequency spectra, (**d**) the vibration signal from the accelerometer and frequency spectra.

**Figure 12:**
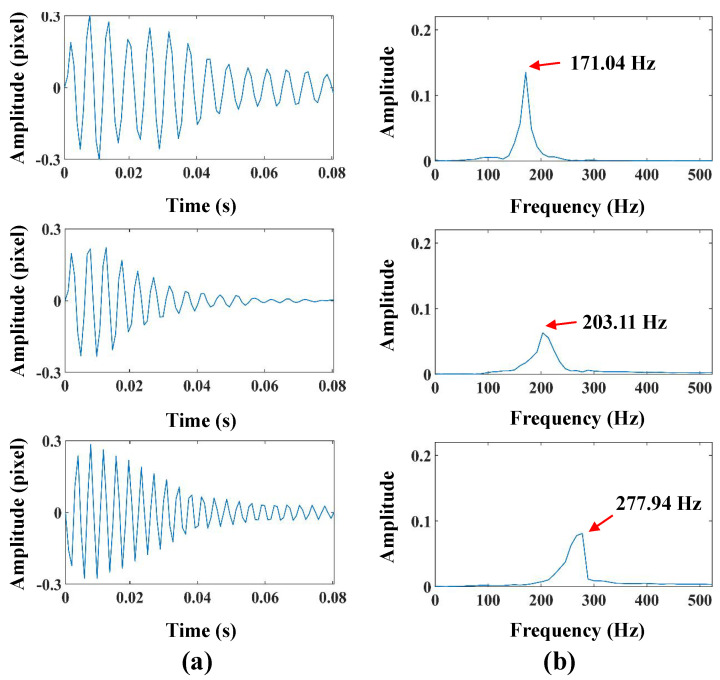
(**a**,**b**), the first three orders of modal responses and frequency spectra in the arch dam model test.

**Figure 13:**
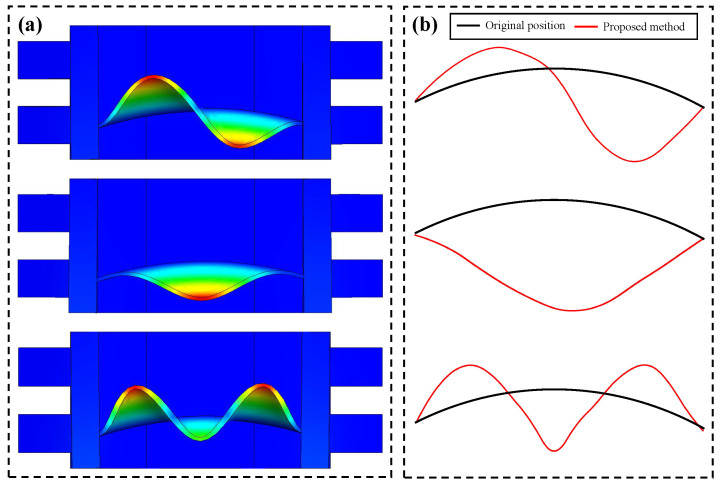
(**a**,**b**) Comparison results of modal shapes obtained by finite element simulation and the proposed method in the arch dam model test.

The colored lines in Figure 11a indicate the extraction results of the dam body pixels, and the yellow line indicates the location of the spatial-temporal slice in the arch dam model test. Figure 11b shows the spatial-temporal slices from bilateral edges. Figure 11c presents the vibration signal from bilateral edges and its frequency spectra. Figure 11d shows the vibration signal from the accelerometer and frequency spectra. In this case, the running time of the sampling and vibration extraction processes is slightly longer than that in the beam test (41.3 s). After separation by VMD, the first three orders of modal responses were reserved. The separated time-domain modal responses and frequency spectra are illustrated in Figure 12. Three obvious peaks, including 171.04 Hz, 203.11 Hz, and 277.94 Hz, were detected from frequency spectra. The analysis results of finite element simulation and experimental modal frequencies in the arch dam model test are listed in Table 4. Moreover, Figure 13a,b illustrate the comparison of modal shapes obtained by finite element simulation and the proposed method in the arch dam model test.

## 4. Discussion

The experiments on two typical structures validate the effectiveness of the proposed method. Using an efficient process, the extraction of vibration signals in these two cases is controlled within 1 min. The only step that requires manual intervention is the estimation of modal responses (setting the parameters in VMD). Without the interface of spatial filters, the proposed method only focuses on the dynamic deviations on edge profiles instead of all pixel coordinates. The modal shapes are observed by estimating spatial weights along the edges instead of manipulating global spatial information. These advantages make the method more efficient in practical applications. Similar to the deviation magnification [30], the direction of temporal samplings in the proposed method should also be along the normal line of curve edges. For more complex curve edges, the critical problem in temporal sampling is to locate edge coordinates and find their normal directions. A promising solution to the problem is to match these curves with appropriate equations and then calculate the sampling directions in space. However, the error in curve fitting will affect the accuracy of vibration signals. This is a common problem, and its solution has significance for the practical extension of both the deviation magnification algorithm and the proposed method.

## 5. Conclusions

This paper proposes an efficient method for extracting structural vibration signals and visualizing modal shapes by processing spatial-temporal slices on structural edges. In the proposed method, the perspective regarding deviation changes from space to the timeline. Meanwhile, by temporal samplings on all edge positions, dynamic deviations of these datasets can accurately reveal vibrations on structural edges. Based on the assumption of a linear vibration system, the modal shapes are observed by calculating the spatial weights corresponding to a specific mode. During the processing, the VMD and MAF algorithms are employed, respectively, to calculate modal responses and refine the estimated spatial weights. Finally, the effectiveness of the proposed method is verified by a simulation test and two laboratory experiments on typical structures. The analysis results have proven the accuracy and efficiency of the presented processing flow. In the discussion part, the importance of sampling for both the proposed method and the deviation magnification is emphasized. In future work, we will further investigate the automation of the positioning of edges and normal direction coordinates, as well as the feasibility of applying the proposed method to real scale structures.

## Figures and Tables

**Table 1:** The technical information about the high-speed camera.

Model	Sensor	Maximum Resolution	FPS at Maximum Resolution	Memory Capacity	Shutter
Chronos 2.1-HD	CMOS	1280 × 1024 pixels	1069 fps	32 GB	Global shutter

**Table 2:** The parameters of the beam and analysis of the modal frequencies in the beam test.

Young’s Modulus (GPa)	Density (kg · m${\;\;}^{-3}$)	Mode Order	Simulation (Hz)	Experiment (Hz)	Error (%)
		1	15.47	15.50	0.19
72	$2.66\times 10^{3}$	2	96.87	98.88	2.07
		3	271.16	279.54	3.09

**Table 3:** Comparison of the cross-MAC values of the modal shapes obtained by finite element simulation and the modal shapes obtained by the proposed method.

	Proposed Method		
	Mode 1	Mode 2	Mode 3
Finite element simulation	0.9971	0.9524	0.9887

**Table 4:** The analysis results of finite element simulation and experimental modal frequencies in the arch dam model test.

Mode Order	Simulation (Hz)	Experiment (Hz)	Error Rate (%)
1	169.82	171.04	0.72
2	206.39	203.11	1.59
3	284.95	277.94	2.46

## Data Availability

Data will be made available on request.

## References

[1] Delgadillo R.M., Casas J.R. (2022). Bridge damage detection via improved completed ensemble empirical mode decomposition with adaptive noise and machine learning algorithms. Struct. Control. Health Monit..

[2] Wada D., Igawa H., Kasai T. (2016). Vibration monitoring of a helicopter blade model using the optical fiber distributed strain sensing technique. Appl. Opt..

[3] Hızal Ç. (2021). Frequency domain data merging in operational modal analysis based on least squares approach. Measurement.

[4] Xue Z.-P., Wang C., Li M., Wang C., Jia H., Yu Y. (2021). Image-based method for the angular vibration measurement of a linear array camera. Appl. Opt..

[5] Xu Y., Brownjohn J.M., Hester D. (2019). Enhanced sparse component analysis for operational modal identification of real-life bridge structures. Mech. Syst. Signal Process..

[6] Altunişik A.C., Günaydin M., Sevim B., Bayraktar A., Adanur S. (2016). Dynamic Characteristics of an Arch Dam Model before and after Strengthening with Consideration of Reservoir Water. J. Perform. Constr. Facil..

[7] Sheng Z., Chen B., Hu W., Yan K., Miao H., Zhang Q., Yu Q., Fu Y. (2021). LDV-induced stroboscopic digital image correlation for high spatial resolution vibration measurement. Opt. Express.

[8] Zhang W., Tao Y., Wu Y., Zhu F., Cai W., Liu N., Zhao Q., Xue P. (2023). Vibration measurement with frequency modulation single-pixel imaging. Chin. Opt. Lett..

[9] Feng D., Feng M.Q. (2018). Computer vision for SHM of civil infrastructure: From dynamic response measurement to damage detection—A review. Eng. Struct..

[10] Feng D., Feng M.Q. (2017). Experimental validation of cost-effective vision-based structural health monitoring. Mech. Syst. Signal Process..

[11] Cakar O., Sanliturk K. (2005). Elimination of transducer mass loading effects from frequency response functions. Mech. Syst. Signal Process..

[12] Wang X., Li F., Du Q., Zhang Y., Wang T., Fu G., Lu C. (2023). Micro-amplitude vibration measurement using vision-based magnification and tracking. Measurement.

[13] Khuc T., Nguyen T.A., Dao H., Catbas F.N. (2020). Swaying displacement measurement for structural monitoring using computer vision and an unmanned aerial vehicle. Measurement.

[14] Zhang D., Hou W., Guo J., Zhang X. (2021). Efficient subpixel image registration algorithm for high precision visual vibrometry. Measurement.

[15] Wang J., Zhao W., Leach R., Xu L., Lu W., Liu X. (2021). Positioning error calibration for two-dimensional precision stages via globally optimized image registration. Measurement.

[16] Dai X., Qi J., Xu M., Zhang W., Wang Y., Zhang J., Yang W. (2023). Omnidirectional 3D-DIC method for determining inner deformation in pipelines. Measurement.

[17] Cofaru C., Philips W., Paepegem W.V. (2012). A three-frame digital image correlation (DIC) method for the measurement of small displacements and strains. Meas. Sci. Technol..

[18] Reu P.L., Rohe D.P., Jacobs L.D. (2017). Comparison of DIC and LDV for practical vibration and modal measurements. Mech. Syst. Signal Process..

[19] Wadhwa N., Rubinstein M., Durand F., Freeman W.T. (2013). Phase-Based Video Motion Processing. ACM Trans. Graph..

[20] Wadhwa N., Wu H.Y., Davis A., Rubinstein M., Shih E., Mysore G.J., Chen J.G., Buyukozturk O., Guttag J.V., Freeman W.T. (2016). Eulerian Video Magnification and Analysis. Commun. ACM.

[21] Southwick M., Mao Z., Niezrecki C. (2021). Volumetric Motion Magnification: Subtle Motion Extraction from 4D Data. Measurement.

[22] Wu H.Y., Rubinstein M., Shih E., Guttag J., Durand F., Freeman W.T. (2012). Eulerian Video Magnification for Revealing Subtle Changes in the World. ACM Trans. Graph. (Proc. Siggraph 2012).

[23] Cho D., Gong J. (2023). A Feasibility Study on Extension of Measurement Distance in Vision Sensor Using Super-Resolution for Dynamic Response Measurement. Sensors.

[24] Chen Z., Ruan X., Zhang Y. (2023). Vision-Based Dynamic Response Extraction and Modal Identification of Simple Structures Subject to Ambient Excitation. Remote. Sens..

[25] Lei X., Jin Y., Guo J., Zhu C. (2015). Vibration extraction based on fast NCC algorithm and high-speed camera. Appl. Opt..

[26] Sarrafi A., Mao Z., Niezrecki C., Poozesh P. (2018). Vibration-based damage detection in wind turbine blades using Phase-based Motion Estimation and motion magnification. J. Sound Vib..

[27] Chen J.G., Wadhwa N., Cha Y.J., Durand F., Freeman W.T., Buyukozturk O. (2015). Modal identification of simple structures with high-speed video using motion magnification. J. Sound Vib..

[28] Zhang D., Zhu A., Wang Y., Guo J. (2022). Hybrid-driven structural modal shape visualization using subtlevariations in high-speed video. Appl. Opt..

[29] Zhang Q., Su X. (2005). High-speed optical measurement for the drumhead vibration. Opt. Express.

[30] Wadhwa N., Dekel T., Wei D., Durand F., Freeman W.T. (2015). Deviation Magnification: Revealing Departures from Ideal Geometries. ACM Trans. Graph..

[31] Dragomiretskiy K., Zosso D. (2014). Variational Mode Decomposition. IEEE Trans. Signal Process..

[32] Hong N., Tang C., Xu M., Lei Z. (2022). Phase retrieval for objects in rain based on a combination of variational image decomposition and variational mode decomposition in FPP. Appl. Opt..

[33] Mazzeo M., De Domenico D., Quaranta G., Santoro R. (2023). Automatic modal identification of bridges based on free vibration response and variational mode decomposition technique. Eng. Struct..

[34] Chang Z., Gao Q., Monti G., Yu H., Yuan S. (2023). Selection of pulse-like ground motions with strong velocity-pulses using moving-average filtering. Soil Dyn. Earthq. Eng..

[35] Harmanci Y.E., Gülan U., Holzner M., Chatzi E. (2019). A Novel Approach for 3D-Structural Identification through Video Recording: Magnified Tracking. Sensors.

[36] Sevim B., Bayraktar A., Altunişik A., Adanur S., Akköse M. (2012). Determination of water level effects on the dynamic characteristics of a prototype arch dam model using ambient vibration testing. Exp. Tech..

[37] Gomes J.P., Lemos J.V. (2020). Characterization of the dynamic behavior of a concrete arch dam by means of forced vibration tests and numerical models. Earthq. Eng. Struct. Dyn..

